# Age‐Specific Functional Connectivity Changes After Partial Sleep Deprivation Are Correlated With Neurocognitive and Molecular Signatures

**DOI:** 10.1111/cns.70272

**Published:** 2025-02-11

**Authors:** Liyong Yu, Xuanyi Chen, Yuqi He, Xiaojuan Hong, Siyi Yu

**Affiliations:** ^1^ School of Acupuncture and Tuina Chengdu University of Traditional Chinese Medicine Chengdu China; ^2^ Key Laboratory of Acupuncture for Senile Disease (Chengdu University of TCM) Ministry of Education Chengdu China

**Keywords:** Allen Human Brain Atlas, default mode network, graph theory, Neurosynth, sleep deprivation

## Abstract

**Background:**

This study aimed to investigate age‐specific alterations in functional connectivity after sleep deprivation (SD) and decode brain functional changes from neurocognitive and transcriptomic perspectives.

**Methods:**

Here, we examined changes in global and regional graph measures, particularly regional network strength (RNS), in 41 young participants and 36 older participants with normal sleep and after 3 h of SD. Additionally, by utilizing cognitive probabilistic maps from Neurosynth and gene expression data from the Allen Human Brain Atlas, we applied partial least‐squares regression analysis to identify the neurocognitive and transcriptional correlates of these RNS changes.

**Results:**

After SD, older participants exhibited decreased RNS in the default mode network (DMN) and dorsal attention network, with increased RNS in the visual network. Young participants also showed decreased RNS in the DMN, notably in the left inferior parietal lobe, left dorsolateral prefrontal cortex, and left posterior cingulate cortex. In young participants, SD‐induced RNS changes significantly correlated with cognitive processes such as “attention,” “cognitive control,” and “working memory,” while in older participants, they correlated with “learning,” “focus,” and “decision.” Gene‐category enrichment analysis indicated that specific genes related to signal transduction, ion channels, and immune signaling might influence SD pathophysiology by affecting functional connectivity in young participants.

**Conclusions:**

This study elucidates shared and age‐specific brain functional network alterations associated with SD, providing a neurocognitive and molecular basis for understanding the underlying pathophysiology.

## Graphical abstract

This study found shared and age‐specific brain functional network alterations after sleep deprivation compared to normal sleep conditions. Additionally, these age‐specific abnormal connectivity patterns correlate with the spatial distribution of specific cognitive processes and specific genes related to signal transduction, ion channels, and immune signaling.

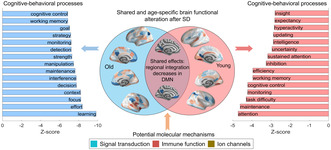

## 1 Introduction

Sleep deprivation (SD) is increasingly common in our society and can affect multiple levels of brain organization [1]. At the macroscope level, SD impacts cognitive‐behavioral functions, including mood [2], attention, and memory [3], and those dysfunctions were found to be associated with brain network alterations [4]. At the microscope level, molecular processes such as immune response [5], systemic inflammation [6], and calcium signaling [7] were found to be associated with SD, and these processes in specific brain regions may mediate the cognitive deficits in people with insufficient sleep [8]. The identification of cortical regions affected by SD and the comprehension of the resultant cognitive‐behavioral dysfunctions are crucial [9]. Moreover, exploring the link between disrupted brain network connectivity and molecular processes may yield valuable understandings of the pathological mechanism of SD.

Functional magnetic resonance imaging (fMRI) detects brain activity by measuring blood oxygen changes, allowing visualization of whole‐brain networks. The brain's functional organization can be studied using methods like seed‐based correlation, independent component analysis, and graph theory [10]. In graph theory, the brain is modeled as a network of nodes (brain regions) and edges (connections between nodes), with various measures used to quantify and describe these networks [11]. Changes in these graph measures, reflecting shifts in functional connectivity, have been identified as key indicators in neurological disorders [12], underscoring the importance of understanding how SD impacts brain connectivity.

Studies combining MRI and graph theory in individuals with SD have revealed abnormalities in the brain's topological efficiency. For instance, a task‐based fMRI study demonstrated that SD alters the regional architecture of the default mode and sensorimotor networks [13]. Additionally, a recent diffusion tensor imaging study found that individuals vulnerable to SD exhibited lower efficiency in both global and regional graph measures compared to those resistant to SD after 24 h of deprivation [14]. Limited studies [15] have yet explored the impact of partial sleep deprivation, which is a more ecologically relevant condition compared to total sleep deprivation. Previous research also suggests that resistance to SD is more common in older adults [16, 17], likely due to the higher prevalence of sleep disturbances and a reduced impact of sleep restriction on cognitive outcomes in this age group [16, 18]. However, the effects of short‐term (3‐h) SD on resting‐state fMRI (rs‐fMRI) topological efficiency across different age groups (young and old) remain unclear.

While the above macroscopic insights into susceptible brain regions are invaluable, the cognitive dysfunctions and molecular mechanisms underlying SD‐related brain functional abnormalities remain largely unexplored. Advances in technology and data sharing now offer new opportunities to link these topological changes to molecular dynamics and psychological processes [19]. For instance, high‐resolution functional neuroimaging has enabled the creation of comprehensive meta‐analytical atlases, illustrating how specific brain areas respond to a wide range of perceptual, cognitive, and affective manipulations [20, 21]. Concurrently, high‐throughput microarray profiling has produced detailed gene expression maps across the brain [22, 23], facilitating inferences about the spatial distribution of molecular processes and cell types [24, 25, 26]. Together, these global initiatives in functional mapping and brain genomics provide an unprecedented opportunity to identify the neurocognitive and transcriptional correlates of SD‐induced connectivity changes.

**FIGURE 1:**
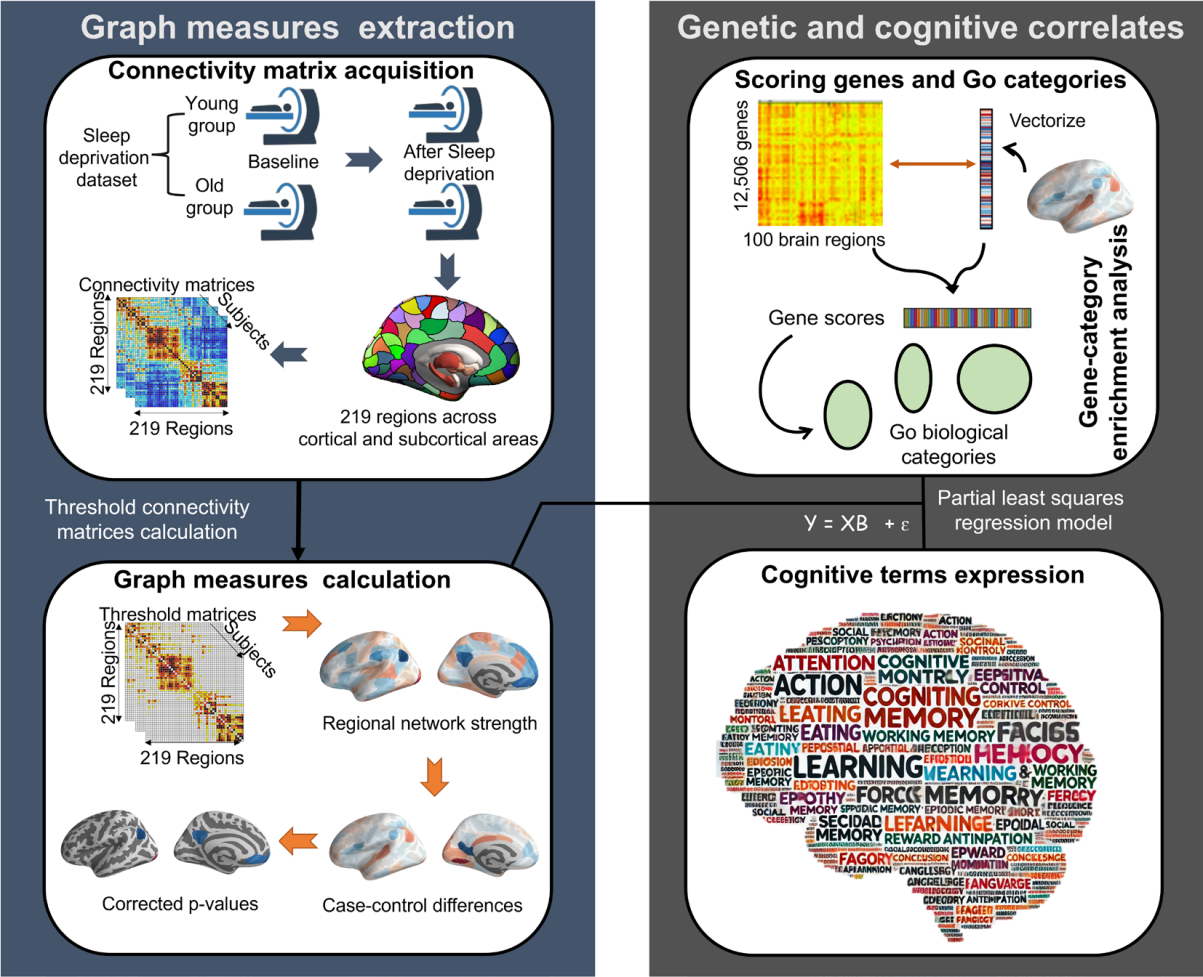
Schematic summary of the study design. The workflow is split into a graph measures extraction module and a context module for contextualizing results with external datasets.

In this study, we focused on identifying brain regions that characterize functional networks after SD across different age groups and decoding these topological changes through microscale transcriptomic data and macroscale psychological processes. The framework of the study consisted of several steps (Figure 1). First, we assessed whether global and regional topological efficiency of the brain changes after partial SD in both young and older adults. We then applied partial least‐squares regression (PLS‐R) analysis [4] to decode the SD‐induced regional topological changes using gene expression data from the Allen Human Brain Atlas [22] and cognitive probabilistic terms from Neurosynth [21].

## 2 Materials and Methods

### 2.1 Participants

This study involved 86 participants from the previous Stockholm Sleepy Brain Study, focusing on the effects of sleep deprivation across different age groups [27, 28]. Detailed information on the sample, including inclusion and exclusion criteria, is available in the original publications [15, 27, 28] and in our Appendix S1. The full sample consisted of 47 younger (20–30 years old) and 39 older (65–75 years old) participants. As described in previous study [15, 27, 28], healthy volunteers underwent MRI scanning on two occasions approximately 1 month apart after normal sleep (NS) and partial SD in a counter‐balanced and randomized fashion. Participants were instructed to sleep as usual in the NS condition. In the SD condition, participants were instructed to sleep 3 h in the end of their normal sleep period.

### 2.2 MRI Data Acquisition

As described in previous study [27], T1‐weighted (T1w) and rs‐fMRI images were acquired using a General Electric Discovery MR750 3T MRI scanner with an 8‐channel head coil. The following settings were used to acquire sagittal 3D T1w images: repetition time (TR) = 6.4 ms; echo time (TE) = 2.81 ms; flip angle (FA) = 11°; acquisition matrix = 512 × 512; slice thickness = 1 mm, no gap; and 180 sagittal slices. The rs‐fMRI images were obtained with the following parameters: TR = 2500 ms; TE = 30 ms; FA = 75°; acquisition matrix = 128 × 128; voxel size = 2.25 × 2.25 × 3.0 mm^3^; thickness = 3.0 mm; and number of slices = 49.

### 2.3 MRI Data Preprocessing and fMRI Connectivity Features Extraction

The preprocessing of T1w and rs‐fMRI data was performed on fMRIPrep 23.1.1 pipelines [29] (https://fmriprep.org). For the T1w image, a sequence of procedures was conducted, which encompassed intensity non‐uniformity correction, skull‐stripping, tissue segmentation, and spatial normalization to a standard space through nonlinear registration. The fMRI preprocessing procedures encompassed reference volume generation, head‐motion parameter estimation prior to spatiotemporal filtering, slice‐time correction, co‐registration with the T1w reference, extraction of confounding timeseries, and resampling into standard space. Additionally, fMRIPrep was used to estimate 36 confounds from the preprocessed time points [30, 31]. These confounding matrices were utilized within xcp_d 0.0.4 [31, 32] (https://xcp‐d.readthedocs.io) to mitigate motion‐related artifacts and noise in rs‐fMRI data. The timeseries were band‐pass filtered (0.01–0.1 Hz) and the denoised timeseries was smoothed using Nilearn with a Gaussian kernel (FWHM = 6.0 mm). Framewise displacement (FD) was calculated across the entire time point for each subject as head movement measurements. Processed functional time series were then extracted from Schaefer 200 cortical areas [33] and 19 subcortical regions [34]. Additionally, we employed the Yeo 7‐network parcellation [35] to assign each cortical ROI to a corresponding functional network (https://github.com/PennLINC/AtlasPack/blob/main/Schaefer/atlas‐Schaefer2018v0143_desc‐200ParcelsAllNetworks_dseg.tsv). In cases of partial coverage for each parcel, uncovered voxels (values of all zeros or NaNs) were either ignored (when the parcel had > 50.0% coverage) or were set to zero (when the parcel had < 50.0% coverage). Then pairwise functional connectivity was computed, operationalized as Pearson's correlation of smoothed time series from each parcel, resulting in a 219 × 219 connectivity matrix. After excluding the first ten non‐steady‐state volumes of the BOLD data, subjects were excluded if they had more than 20% time point with 0.5 mm movement based on FD. We then investigated the within‐group differences in connectivity matrices for both young and old participants after SD. For each group, we compared the fMRI connectivity values of each undirected pair using 47,742 two‐sided paired *t*‐tests per connectivity matrix. The significance threshold was set at *p* < 0.001.

### 2.4 Graph Measures Extraction

For each 219 × 219 connectivity matrix, the analysis employed thresholded and binarized connectivity matrices, retaining the top 20% strongest connections, a threshold known for delivering reproducible graph measures based on connectivity analysis [36].

The regional network measure was calculated as the regional network strength (RNS) to assess regional connectedness [37]. The RNS refers to the number of connections a node has within the thresholded network. The global network measures computed were the global clustering coefficient, global efficiency, and smallworldness [37]. The global clustering coefficient, an indicator of functional segregation, is the average clustering coefficient of all nodes. Global efficiency, reflecting functional integration, is the average of the inverse shortest path length between all node pairs, indicating efficient information transfer between distant regions. Smallworldness compares the ratio of functional integration to segregation against a random network of the same size and degree, characterizing networks that are highly clustered with short characteristic path lengths [38]. Both regional and global network measures were calculated using Brain Connectivity Toolbox [12] (http://www.brain‐connectivity‐toolbox.net).

### 2.5 Effects of SD in Graph Network Measures

For regional graph measures statistical analysis, we utilized paired *t*‐tests to estimate the within‐group differences before and after SD in the RNS across two different groups (old and young). In adjusting the *p*‐values obtained for 219 regions to reduce false positives, we employed the False Discovery Rate (FDR) correction method. After conducting multiple comparisons (*n* = 219), the significance threshold was set at *p*_FDR_ < 0.05 to ensure the statistical validity of the results. Additionally, similar paired *t*‐tests were conducted to investigate the within‐group differences associated with SD in three global network measures across two groups (old and young). FDR correction was also used to correct multiple comparisons (*n* = 3).

### 2.6 Cognitive Processes‐Cortical Regional Network Strength Spatial Correspondence Analysis

We obtained probabilistic measures of the association between cortical areas and cognitive processes from Neurosynth, a meta‐analytic tool synthesizing results from more than 15,000 published functional MRI studies [21] (https://neurosynth.org/). Although Neurosynth includes over 1000 cognitive processes, our focus was specifically on 123 neurocognitive processes related to cognitive and behavioral functions [39]. The coordinates of each term reported by Neurosynth were parcellated according to the Schaefer 200 atlas [33]. Those 123 neurocognitive terms across the 200 cortical areas can be found in the Appendix S1. These probabilistic measures can be interpreted as a quantitative representation of how regional neural activity variations align with psychological processes [4]. We then applied PLS‐R [40] to relate disparities in the RNS patterns identified by paired *t*‐tests (response variables, represented by the paired *t* value) to cognitive functions (predictor variables, represented by the 123 selected terms) [19, 41]. The PLS‐R was conducted for the old and young groups separately. Out of 1–15 possible components, we focused on the one exhibiting the most substantial variance in PLS‐R analysis, consistently finding it to be the first component (PLS1). The significance of the variance explained by PLS1 was verified using spatial permutation of the response variables to generate a null distribution for comparison (see Spatial Null Models section for details). We computed the *Z*‐scores for each term using bootstrapping procedure [24] as their contribution to PLS1.

### 2.7 Gene‐Cortical Regional Network Strength Spatial Correspondence Analysis

We initially delineated normative gene expression patterns from the Allen Human Brain Atlas (AHBA) [22], and mapped these profiles onto the Schaefer 200 functional parcellation [33]. Due to limited right hemisphere data (only two participants), the transcriptomic‐imaging analysis focused on the left hemisphere [24, 42], represented by a 100‐region × 12,506‐gene matrix. Those 12,506 genes in the 200 cortical areas can be found in the Appendix S1. PLS‐R [43] was then used to assess the covariance between gene expression patterns (predictor variables, represented by the 100 × 12,506 matrix) and RNS changes (response variables, represented by the paired *t* value) in the left hemisphere separately for the old and young groups. The PLS1 represented a linear composite of gene expression scores, weighted to reflect their collective covariance with alterations in RNS most closely. A similar spatial permutation test was used to verify the significance of PLS1 to reduce false positives, and a bootstrapping procedure was employed to calculate the *Z*‐scores for each gene as their contributions to the PLS1 [40]. Furthermore, to interpret PLS results, we used gene‐category enrichment analyses [44] to understand which molecular pathways were enriched among the most highly associated genes. For each significant PLS model, the score for an enriched category was calculated as the mean gene *Z*‐score in the bootstrapping procedure within the category. A spatial permutation test was also conducted to minimize false positives for each enriched category. Further details are provided in the Appendix S1.

### 2.8 Statistical Analysis

Data analysis was carried out using MATLAB 2022b (The MathWorks Inc., Natick, MA, USA), applying a significance level of *p* < 0.05. To assess the normality of the imaging metrics, the Shapiro–Wilk test was employed. The statistical significance of within‐group differences was evaluated using paired *t*‐tests for normally distributed data, and paired Wilcoxon tests for non‐normally distributed data. However, it was crucial for our analysis to correlate values from SD‐induced brain functional changes with those from brain regions in other atlases. To facilitate this, we utilized paired *t*‐tests for the graph measures to obtain continuous *t*‐values, thereby minimizing numerical heterogeneity from different tests. Additionally, we performed a two‐sample *t*‐test to examine any between‐group differences in regional and global graph measures at baseline and after SD. For both regional and global network graph metrics, FDR correction was applied to account for multiple comparisons. Moreover, it is advisable to test the reliability of results with varying binarizing thresholds [45] in graph measure extraction. To validate our main results, we tested alternative thresholds (10%, 30%, 40%) to confirm the robustness of RNS and global graph measure changes in the young group.

### 2.9 Spatial Null Models

To validate the statistical significance of the behavioral‐neuroimaging and gene‐neuroimaging correlations revealed by the PLS‐R models, we compared the real‐data‐derived PSL1 against null distributions. These distributions were generated from 10,000 spatial permutations (the “spin test”). This permutation involved randomly rotating the neuroimaging vector rows based on a spherical projection of the cortical surface [46]. The spatial permutation technique is available at https://github.com/frantisekvasa/rotate_parcellation. This method ensures the preservation of the inherent correlational structure of the cortical surface data, offering stringent control against false positives in contrast to conventional random permutation tests [46]. This model was used to facilitate the examination of the null PLS1 variance against the actual PLS1 variance derived from the original data. A PLS1 explained variance exceeding the 95th percentile in the distribution of the explained variance from the spatially rotated (*p*_spin_) null models was considered statistically significant. Since subcortical regions lack a spherical structure, the “spin test” cannot be technically applied to them. Therefore, it is only suitable for cortical areas in our study. Additionally, for the correlation analysis between two cortical maps, we also conducted spatial‐null models to reduce spatial auto‐correlations. This method compares the empirical correlation between two spatial maps to a set of null correlations. Each correlation between two cortical maps is reported with a *p*‐value from spherical permutation (*p*_spin_), derived from comparing the actual Spearman Rho against a null distribution of 10,000 correlations, using one real map and rotated projections of the other.

## 3 Results

### 3.1 Participants

The initial sample included 47 younger and 39 older participants. However, due to dropouts, fMRI preprocessing failures, and excessive movement during scanning, the final analysis included 41 younger and 36 older participants. As previously reported [15, 27, 28], SD significantly increased self‐rated sleepiness (Table S1).

### 3.2 Partial Sleep Deprivation Effects on Connectivity Measures Across Old and Young Group

**FIGURE 2:**
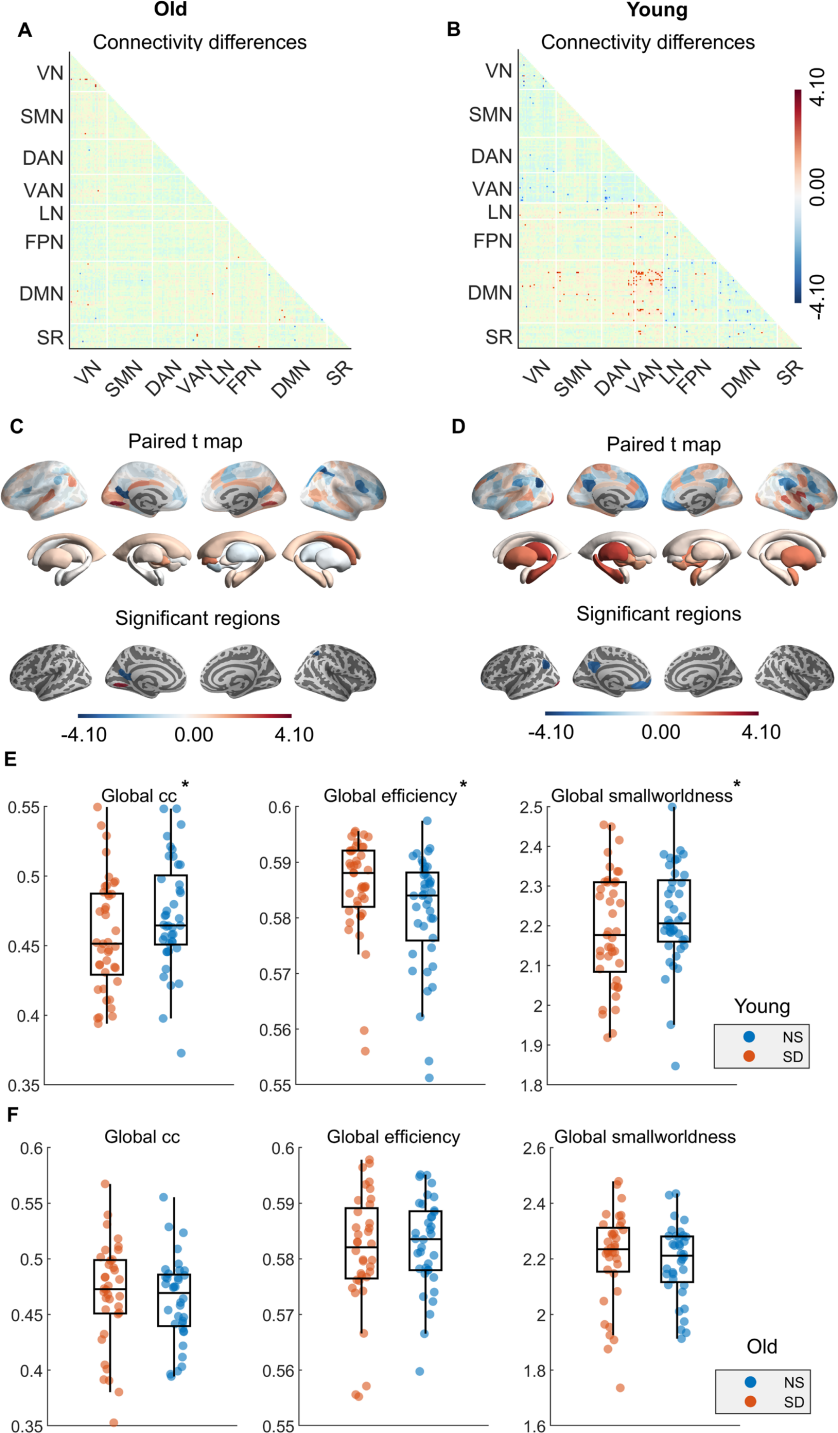
Within‐group differences in resting state fMRI across old and young participants. (A, B) Within‐group differences in pairwise connectivity across the old and young groups. Blue values indicate lower connectivity after SD. All connections not showing strong evidence (*p* < 0.001) are faded out. (C, D) Within‐group differences in the regional network strength (RNS) across the old and young groups. Regions with blue color indicate lower RNS after SD, and red indicate higher RNS after SD. Only locations with strong evidence (*p*_FDR_ < 0.05) for a difference in the RNS are depicted as significant. The detailed information of significant regions can be found in Table S2. (E) Within‐group differences of the global graph measures in the young group. (F) Within‐group differences of the global graph measures in the old group. *p_FDR_ < 0.05. cc, clustering coefficient; DAN, dorsal attention network; DMN, default mode network; FPN, fronto‐parietal network; LN, limbic network; NS, normal sleep; SD, sleep deprivation; SMN, somato‐motor network; SR, subcortical regions; VAN, ventral attention network; VN, visual network.

We first examined whether partial SD induced differences in the connectivity matrices within young and older adult groups. For pairwise connectivity measures, *t*‐values are color‐coded to indicate the direction of effects, with statistically significant results (Figure 2A,B; *p* < 0.001) highlighted. An increase in connectivity was observed in young participants after SD, primarily between the default mode network (DMN) and the salience‐ventral attention network, a pattern not evident in the older participants.

Next, we analyzed within‐group differences in graph measures after SD. For local graph measures, positive *t*‐values indicate higher RNS after SD compared to NS, while negative *t*‐values indicate lower RNS. In older participants, RNS decreased in regions within the DMN and dorsal attention network but increased in the visual network (*p*_FDR_ < 0.05, Figure 2C, Table S2). In the young group, a similar decrease in the DMN was observed, with a more pronounced effect in regions such as the left inferior parietal lobe (IPL), the left dorsolateral prefrontal cortex (DLPFC), and the left posterior cingulate cortex (PCC) (*p*_FDR_ < 0.05, Figure 2D, Table S2). Those changes in RNS for the young group were validated using alternative thresholds (10%, 30%, 40%), with consistent paired *t*‐values observed across 219 cortical and subcortical areas, as compared to the 20% threshold used in the current results.

Regarding global measures, significant within‐group differences were primarily observed in the young group after SD (*p*_FDR_ < 0.05, Figure 2E). The young participants exhibited a significant decrease in the global clustering coefficient and small‐worldness, along with an increase in global efficiency. Those changes in global measures were confirmed using alternative thresholds (10%, 30%, 40%) during graph measure extraction (*p*_FDR_ < 0.05, Figure S1) in the young group. No significant within‐group differences in global network measures were found in the older group (*p*_FDR_ > 0.05, Figure 2F). No significant between‐group differences in regional or global network measures were observed at baseline or after SD (*p*_FDR_ > 0.05, Table S3).

### 3.3 Cognitive‐Behavioral Processes Aligned With SD‐Induced RNS Changes

**FIGURE 3:**
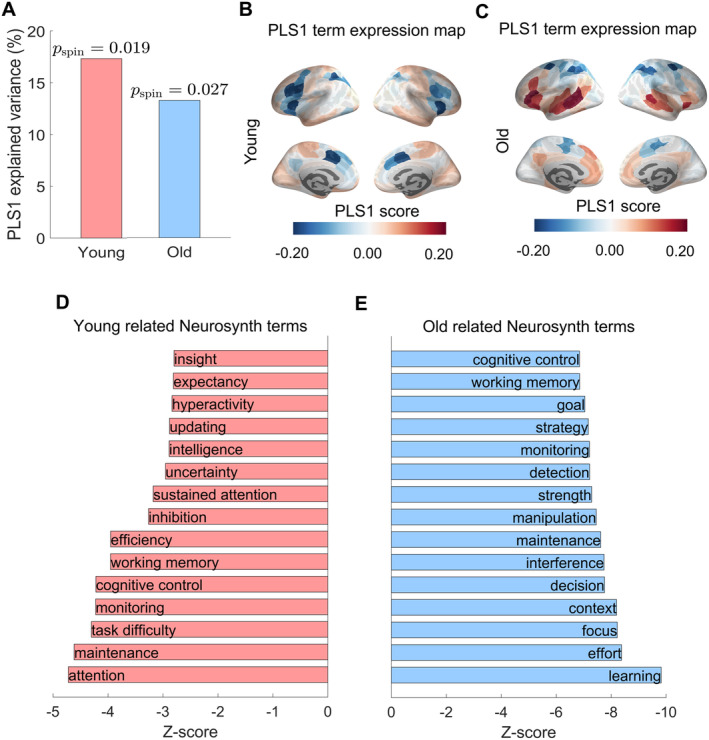
Mapping with‐group differences in RNS to cognitive function. (A) The histogram showed that the significance of the variance elucidated by PLS1 was verified through a permutation of the response variables. The PLS1 component significantly accounted for the total variance in the RNS differences (*p*_spin_ < 0.05), 16.5% in the young group and 13.6% in the old group. (B, C) The PLS1 term expression map in the young and old groups across the 200 cortical areas. (D, E) The top 15 terms with the largest absolute *Z*‐scores are displayed in the young and old groups. PLS1, the first component in the partial least square's regression; RNS, regional network strength.

To associate the SD‐induced RNS changes with specific cognitive processes, we conducted a PLS‐R analysis to relate within‐group differences of RNS separately for the young and old groups to cognitive‐behavioral functions (123 terms). The PLS1 component significantly accounted for 16.5% of the total variance in SD‐induced RNS changes in the young group (*p*_spin_ = 0.014; Figure 3A,B), whereas the PLS1 component in the old group significantly accounted for 13.6% of the total variance in SD‐induced RNS changes (*p*_spin_ = 0.032; Figure 3A,C). Notably, terms such as “attention,” “cognitive control,” and “working memory” exhibited significant correlations with the SD‐induced RNS changes in the young group (*p*_spin_ < 0.05; Figure 3D), whereas “learning,” “focus,” and “decision” exhibited significant correlations with the SD‐induced RNS changes in the old participants (*p*_spin_ < 0.05; Figure 3E). Interestingly, terms such as “cognitive control,” and “working memory” are both significantly correlated with SD‐induced RNS changes in both groups.

### 3.4 Transcriptional Signatures Underlying SD‐Induced RNS Changes in Young Humans

**FIGURE 4:**
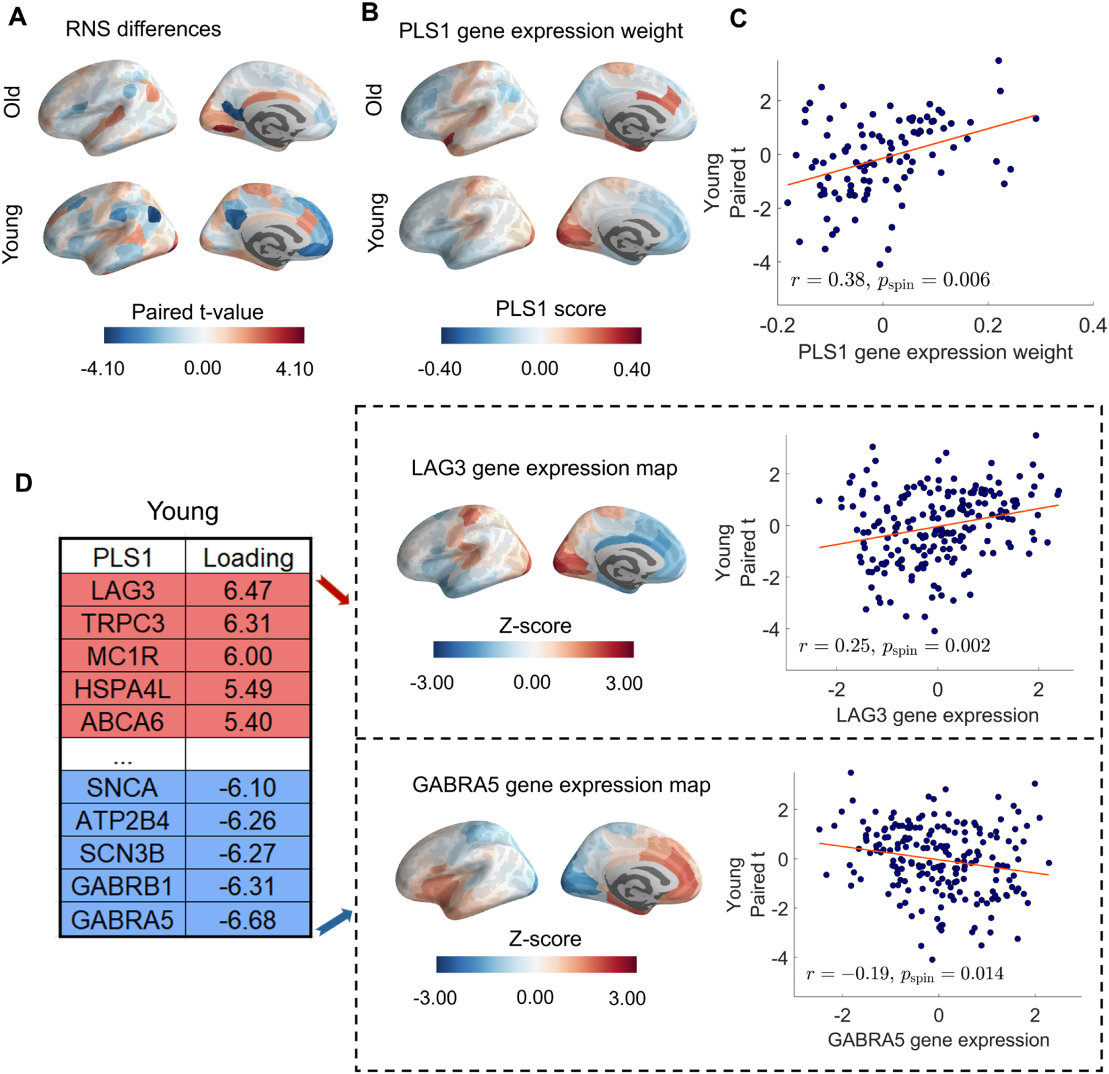
Gene expression profiles related to RNS changes. (A) Cortical map of mean within‐group RNS differences in the left hemisphere. (B) Cortical map of regional PLS1 scores in the young and old group. (C) Scatterplot of regional PLS1 scores (weighted sum of 12,506 gene expression scores) vs. within‐group differences in RNS in the young group (*r* = 0.38, *p*_spin_ = 0.006). (D) Genes that are most positively weighted on PLS1 (i.e., LAG3) correlate positively with SD‐induced RNS differences in the young group (*r* = 0.25, *p*_spin_ = 0.002), whereas genes that are most negatively weighted on PLS1 (i.e., GABAR5) correlate negatively with SD‐induced RNS differences in the young group (*r* = −0.19, *p*_spin_ = 0.014).

Here, we used PLS‐R to pinpoint patterns of gene expression that tracked the spatial distribution of RNS changes after SD (Figure 4A). The PLS1 significantly explained 25.32% of the variations in the macro‐connectivity changes for young participants (*p*_spin_ = 0.004; Figure 4B). However, this variance (12.46%) explained by PLS1 in the old group was not significant after correcting for the spatial permutation test (*p*_spin_ = 0.23). Thus, we only focus on PLS1 in the young group to trace molecular mechanisms. Moreover, a positive correlation was found between PLS1 gene expression scores in 100 cortical regions and paired *t*‐values in reginal RNS in the young group (*r* = 0.51, *p*_spin_ < 0.001, Figure 4C). This positive correlation indicates that genes with positive PLS1 weights are overexpressed in regions with increased RNS after SD, whereas genes with negative PLS1 weights are overexpressed in regions with decreased RNS after SD. Thus, a positive (or negative) PLS1 weight means increased (or decreased) RNS after SD compared to NS, respectively.

**FIGURE 5:**
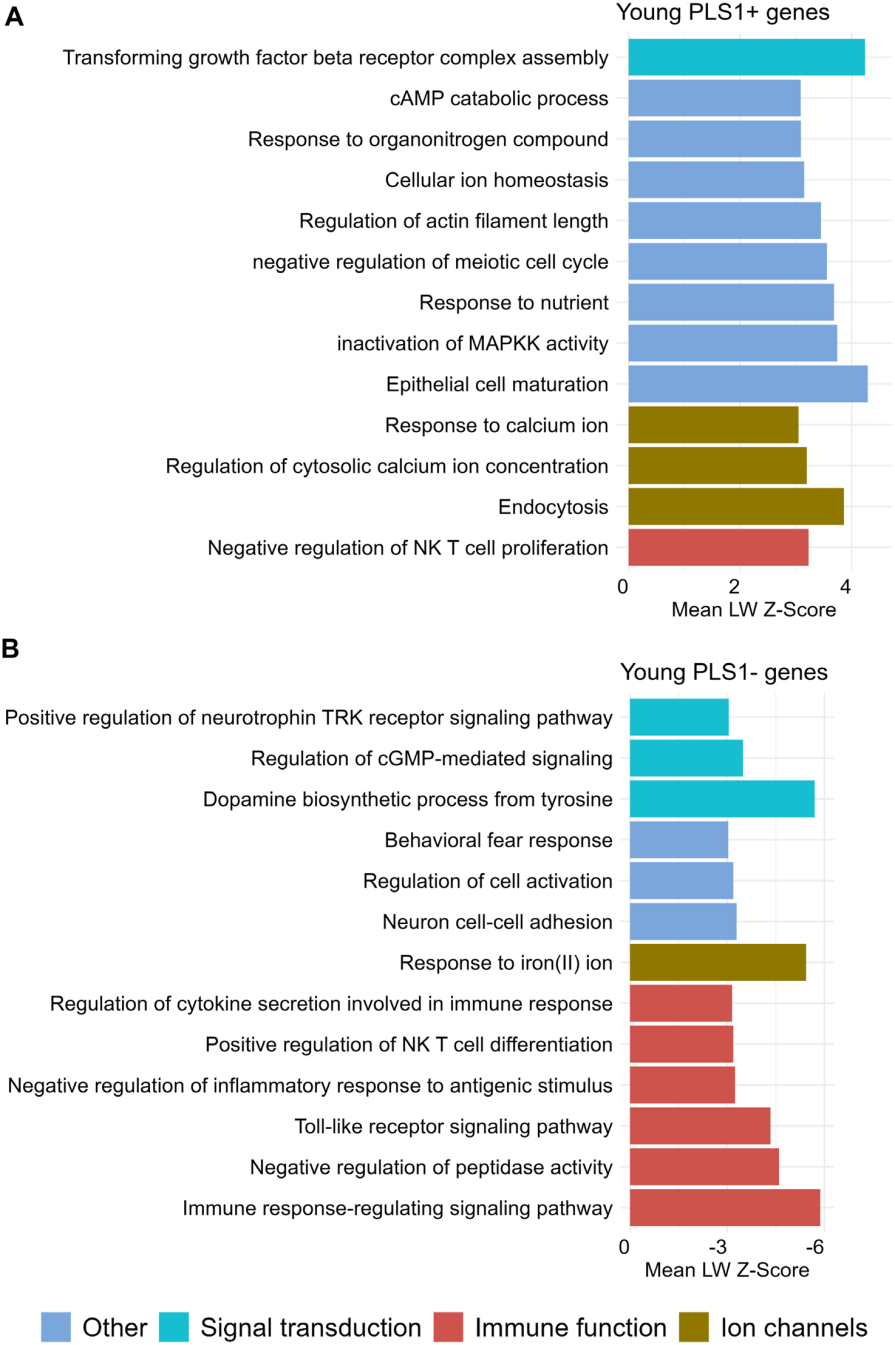
Functional enrichment of PLS1 weighted genes related to RNS effects in the young group. (A) The top 13 significantly biological terms of PLS1+ (*Z*‐score > 3, *p*_spin_ < 0.05). Statistical significance for each term was evaluated compared to shuffled predictor data maintaining spatial autocorrelation (“spin” test, see Methods). (B) The top 13 biological terms in PLS1− (*Z*‐score < −3, *p*_spin_ < 0.05). The term “other” in the figure legend refers to pathways that do not fall under the three primary biological processes identified in our gene‐category enrichment analysis—signal transduction, ion channels, and immune signaling. LW, loading weight; PLS1, the first component in the partial least square's regression.

We identified 910 genes with normalized PLS1 weights *Z* < −3, referred to as the PLS1− gene set, and 744 genes with *Z* > 3, termed the PLS1+ gene set (all *p* < 0.05, FDR corrected, PLS1− and PLS1+ gene lists can be found in the Appendix S1). For example, genes with positive weights included Melanocortin 1 Receptor (MC1R) and ATP Binding Cassette Subfamily A Member 6 (ABCA6), while Synuclein Alpha (SNCA) exhibited negative weights (Figure 4D). To delve deeper into the biological implications of the PLS1+ and PLS1− gene sets, we conducted a gene set enrichment analysis. A subset of the enriched categories, particularly those relevant to immune function, signal transduction, and ion channels, are found to be associated with SD‐induced RNS changes in the young group. Biological processes, such as “response to calcium ion” and “Regulation of cytosolic calcium ion concentration”, are enriched in PLS1+ genes (Figure 5A). The PLS1− gene sets indicate enrichment in processes like “Regulation of cytokine secretion involved in immune response” and “Immune response‐regulating signaling pathway” (Figure 5B).

## 4 Discussions

This study examines neurofunctional changes in SD across different age groups and decodes these alterations from neurocognitive and molecular perspectives. After SD, older participants showed decreased RNS in the DMN and dorsal attention network, with increased RNS in the visual network. Young participants had a similar DMN decrease, notably in the left IPL, left DLPFC, and left PCC. Young participants also showed increased global clustering and small‐worldness, with reduced global efficiency. Moreover, in young participants, SD‐induced RNS changes were significantly correlated with “attention,” “cognitive control,” and “working memory,” while in older participants, they were linked to “learning,” “focus,” and “decision.” Furthermore, PLS‐R identified gene expression patterns significantly associated with SD‐induced RNS changes in young participants, with specific genes associated with signal transduction, ion channels, and immune signaling could influence the pathophysiology by affecting functional connectivity. These findings uncover both shared and age‐specific alterations in SD‐induced functional networks, offering a neurocognitive and molecular basis for understanding the pathophysiology.

Sleep is essential for maintaining health and well‐being, and SD can significantly impair various cognitive functions, including alertness, learning, memory, and executive function [47]. Previous studies have shown that the effects of SD can be observed through alterations in brain functional connectivity networks [13, 14, 48]. Growing evidence suggested that the main effects of SD appear to differ by age group [16, 18]. For example, younger individuals tend to exhibit greater reductions in attention and executive function following SD, while older adults may experience more stable cognitive performance despite sleep restriction [18]. This age‐related resilience or vulnerability to SD highlights the importance of considering age as a modulating factor when examining the impacts of sleep loss on brain connectivity and cognitive function. However, few studies have focused on the topological connectivity alterations to investigate the interaction between aging, SD, and cognitive function.

From our study, we found both shared and age‐specific alterations in SD‐induced functional networks. In terms of pairwise connectivity measures, young participants exhibited increased connectivity between the DMN and the salience‐ventral attention network after partial SD. This change was not observed in older participants. The enhanced connectivity between these networks in younger adults suggests a heightened interaction, possibly reflecting compensatory mechanisms or increased cognitive demands post‐SD [49]. This finding is consistent with previous research suggesting DMN hyperconnectivity in young adults following SD [50]. Age‐related differences in neuroplasticity and compensation likely contribute to this variability in SD effects. Younger individuals, with a generally higher level of neuroplasticity [51], may experience more pronounced connectivity changes as their brains attempt to adapt to the lack of sleep. In contrast, older adults may rely on well‐established, stable neural networks that are less susceptible to short‐term disruptions in sleep [52].

Regarding local graph measures, the RNS refers to the number of connections a node has within the functional network, providing insight into the local connectivity and integration of specific brain regions [53]. While pairwise connectivity measures focus on the direct connectivity between two specific regions, RNS offers a more comprehensive view of how each brain region interacts with the rest of the network [54]. A positive *t*‐value in a node after SD indicated that this region's connectivity within the network increased, suggesting enhanced local integration and potential compensatory mechanisms. In contrast, a negative *t*‐value indicated decreased connectivity, reflecting reduced integration and potential vulnerability to SD effects. In our study, RNS seemed to be more sensitive than the pairwise connectivity measure, as both young and older participants experienced a reduction in RNS within the DMN after SD. Those results indicated a shared impact of SD on the DMN network. However, the effects were more pronounced in younger participants, particularly in regions such as the left IPL, left DLPFC, and left PCC. This suggests that young adults with SD may experience more extensive disruptions in specific brain regions associated with attention and executive functions [55, 56]. Older participants, on the other hand, showed a decrease in RNS within the DMN and dorsal attention network, coupled with an increase in the visual network. The increase in the visual network in older participants after SD may reflect a compensatory shift, where reliance on visual processing becomes more pronounced to maintain cognitive performance [57]. The above results underscore the complexity of SD's impact on local integration of the brain network, reflecting both shared and age‐related effects.

In terms of global measures, significant alterations were observed in the young group after SD. Young participants exhibited a decrease in the global clustering coefficient and small‐worldness, along with an increase in global efficiency. These changes suggest that SD disrupts the balance between network segregation and integration, leading to reduced efficiency [58]. The increase in global measures may reflect a reorganization of network connections to compensate for local disruptions. However, these significant global network changes were not evident in older participants, suggesting age‐specific differences in how SD affects the brain's overall network architecture. The lack of significant global network changes in older adults could imply greater resilience or alternative compensatory mechanisms in their neural networks following SD [18], possibly due to more established neural pathways [13] or different adaptive strategies developed over time [59]. This highlights the importance of considering age when evaluating the impact of SD on brain connectivity [47].

The cognitive dysfunctions frequently linked with SD [60] are reflected in the RNS‐related terms identified in our study. These terms, extrapolated from an aggregation of brain activation data across diverse fMRI studies using Neurosynth [21], highlight the age‐specific and shared cognitive disturbances associated with SD. In young participants, the significant correlation of SD‐induced RNS changes with “attention,” “cognitive control,” and “working memory” suggests that SD primarily affects cognitive functions related to focus and executive processes in this group. These functions are crucial for daily tasks, indicating that young individuals might experience more pronounced impairments in tasks requiring sustained attention and complex problem‐solving following SD [3, 55, 56]. Conversely, older participants exhibited significant correlations with “learning,” “focus,” and “decision,” indicating that SD may impact cognitive processes related to information processing and decision‐making [58]. The shared correlation of “cognitive control” and “working memory” across both groups suggests that these cognitive domains are universally vulnerable to the effects of SD [1, 61], potentially affecting various aspects of cognitive performance regardless of age. Tailoring age‐appropriate sleep management strategies and therapeutic interventions could help mitigate the adverse cognitive impacts of SD, enhancing cognitive resilience in younger individuals and optimizing decision‐making in older adults. These strategies could ultimately improve overall cognitive health across the lifespan.

In young participants, PLS‐R analysis identified gene expression patterns significantly related to SD‐induced RNS changes, explaining 25.32% of the variance. However, this correlation wasn't significant in the older group after correcting for the spatial permutation test (*p*_spin_ > 0.05), indicating that the relationship between gene expression and SD‐induced RNS changes is less pronounced in older adults. This group difference could be due to age‐related changes in brain plasticity and gene expression regulation, which may affect how the brain responds to sleep deprivation at different ages [62]. It highlights the possibility that young and older individuals have distinct biological responses to sleep loss, potentially due to differences in neural adaptability and resilience [18, 59]. Thus, we only focus on PLS1 weighted genes in the young group in our imaging‐transcriptomics analysis. Genes with positive PLS1 weights, such as MC1R and ABCA6, were overexpressed in regions with increased RNS, while negative weights, like SNCA, were linked to decreased RNS. These genes can influence brain function and are crucial in the pathology of SD. For instance, MC1R [63], involved in stress and inflammatory responses, may affect stress regulation and emotional processing, altering cognitive functions under SD. SNCA [64], linked to neurodegenerative diseases, may increase neuroinflammation, leading to decreased RNS and cognitive impairments. These examples highlight the genetic factors influencing SD's effects on brain function.

Based on PLS1 weighted genes, enrichment analysis highlighted associations with signal transduction, ion channels, and immune signaling pathways concerning the SD‐induced RNS changes in the young group. PLS1+ genes were enriched in biological processes such as “response to calcium ion” and “regulation of cytosolic calcium ion concentration.” These processes are crucial for maintaining neuronal excitability and synaptic plasticity [65, 66], which are essential for cognitive functions that are disrupted by sleep deprivation. The enhancement of calcium signaling suggests that increased neuronal activity and excitability in response to SD may contribute to changes in brain network dynamics, potentially leading to cognitive impairments [67]. On the other hand, PLS1− genes were enriched in processes related to “regulation of cytokine secretion involved in immune response” and “immune response‐regulating signaling pathway.” These pathways are indicative of an inflammatory response, which can be exacerbated by SD and may lead to neuroinflammation [68]. The immune signaling pathways may contribute to the disruption of neuronal communication and connectivity [69], resulting in decreased RNS and impaired cognitive functions. The enrichment of these pathways highlights the potential role of neuroinflammation in mediating the adverse effects of SD on brain function. The above understanding of the genetic and molecular underpinnings can help elucidate the complex mechanisms by which SD affects the brain, offering insights into potential therapeutic targets.

Several limitations should be considered. First, we calculated brain functional alterations after only 3 h of SD for each participant. Future research should incorporate different SD durations to verify our findings related to SD‐induced brain changes. Second, our transcriptome analysis was limited to left‐hemisphere regions due to the AHBA database containing right‐hemisphere data for only two subjects [42]. Consequently, our imaging‐transcriptome results for CID may not represent the entire brain. Third, a previous study has shown that individuals vulnerable to SD can recover with normal sleep [70], but it remains unclear if RNS changes during the sleep recovery process. Future longitudinal research should explore whether functional alterations are reversible following SD recovery. Furthermore, although we identified potential neurocognitive and molecular mechanisms associated with RNS alterations, direct causal relationships have not been established and require further investigation.

In conclusion, this study highlights both shared and age‐specific brain functional changes following partial SD. Older participants primarily exhibited RNS alterations in the DMN, whereas young participants showed similar DMN decreases alongside global graph measure changes. Furthermore, imaging‐transcriptomics analysis offers neurocognitive and molecular insights into SD's impact on the brain across different age groups.

## Author Contributions

Siyi Yu and Xiaojuan Hong conceived and designed the study. Xuanyi Chen and Yuqi He performed the study and collected materials. Liyong Yu and Siyi Yu analyzed the results. Liyong Yu, Xuanyi Chen and Yuqi He wrote the manuscript. Siyi Yu and Xiaojuan Hong helped coordinate the study and reviewed the manuscript. All authors contributed to the article and approved the submitted version.

## Conflicts of Interest

The authors declare no conflicts of interest.

## Supporting information


Appendix S1

## Data Availability

Data can be accessed from open repositories in the OpenNeuro (https://openneuro.org/datasets/ds000201/versions/1.0.3).
