# Supplementary material for: Age‐Specific Functional Connectivity Changes After Partial Sleep Deprivation Are Correlated With Neurocognitive and Molecular Signatures
Source: CNS Neurosci Ther. 2025 Feb 11;31(2):e70272. doi: 10.1111/cns.70272 (PMC11811888; doi:10.1111/cns.70272)
Supplement: Supplementary file 1 — Appendix S1 [file CNS-31-e70272-s001.docx]

### 1　|　 Participants

As described in Nilsonne et al. ^1^, participants were recruited by poster advertising on campus sites in Stockholm, on the studentkaninen.se website, and through newspaper ads. Putative participants were screened for inclusion/exclusion criteria using an online form and eligibility was confirmed in an interview upon arrival to the scanning site. Criteria for inclusion were, first, those required to undergo fMRI procedures and to use the hand-held response box, namely: no ferromagnetic items in body, not claustrophobic, not pregnant, no refractive error exceeding 5 diopters, not colorblind, and right-handed. In addition, participants were required to be 20–30 or 65–75 years old (inclusive), to have no current or past psychiatric or neurological illness, including addiction, to not have hypertension or diabetes, to not use psychoactive or immune-modulatory drugs, to not use nicotine every day, and to have a habitual daily caffeine intake corresponding to 4 cups of coffee at most. A further criterion was to not study, have studied, or be occupied in the fields of psychology, behavioral science, or medicine, including nursing and other allied fields. The Insomnia Severity Index (ISI) ^2^, the depression subscale of the Hospital Anxiety and Depression scale (HADS) ^3^ and the Karolinska Sleep Questionnaire (KSQ) ^4^ were used to exclude participants with insomnia symptoms or depression, irregular sleep patterns, or excessive snoring. The HADS scale assesses symptoms of depression and anxiety, and we excluded anyone reporting ≥ 8 on the depression subscale. The ISI measures insomnia symptoms, and we excluded participants rating 15 or above. For KSQ, we excluded all participants reporting snoring or sleep apnea symptoms more than 3 times a week.

### 2　|　 Experimental design

As described in previous study ^1^, healthy volunteers underwent MRI scanning on two occasions approximately one month apart after normal sleep and partial sleep deprivation in a counter-balanced and randomized fashion. In the sleep restriction condition, participants were instructed to sleep 3h in the end of their normal sleep period. As described in Akerstedt et al. ^5^ and in Nilsonne et al. ^1^ polysomnography (PSG) recording took place in the homes of the participants during both the experimental and the control nights, using a solid state, portable sleep recorder (Embla system for the majority of recordings and Vitaport system for a few). Standard electrode (silver/silver chloride) montage for EEG sleep recording was used (C3, C4 referenced to the contralateral mastoid). Sleep staging and respiratory analyses were performed according to the classification criteria of the AASM ^6^ and as implemented in the Siesta group computer assisted scoring procedure ^7^. For analyses of intervention effects, we only included participants who fulfilled previously reported criteria of having slept < 4h in the sleep deprivation condition, and a difference between the two conditions > 2h ^1^. MRI scanning took place in the evening following the sleep intervention and control nights, starting between 5 and 8 p.m. The full sample consisted of 47 younger and 39 older participants (Table S1), but because of dropout after enrolment, data loss (data preprocessing failures, and excessive movement during scanning), and unsuccessful interventions (described in detail in Refs. ^1,8^), the number of participants included for fMRI analyses of the effect of the intervention was 41 younger and 36 older.

### 3　|　Decoding regional network strength changes from molecular level

#### 3.1　|　Estimation of microarray gene expression maps

The AHBA gene expression data ^9^ was processed utilizing the abagen toolbox (version 0.1.3; https://github.com/rmarkello/abagen) ^10^, incorporating steps such as filtering of microarray probes based on intensity, selection of a single probe per gene, matching of samples to brain regions as defined by the 200 functional parcellation ^11^, normalization, and aggregation of data both within and across parcellations. The final gene expression data was represented by a 200 × 15,633 matrix for each donor, relating brain regions to the retained genes. Additionally, genes with low similarity across donors (*r* < 0.2) were removed, resulting in a total of 12,506 genes. As the right hemisphere data was only available for two participants, the transcriptomic-imaging association analysis was limited to the left hemisphere's cortical areas ^12^, represented by a 100-region × 12,506-gene matrix.

#### 3.2　|　Identifying gene expression data correlated with reginal network strength changes

PLS-R was applied to explore the relationship between changes in regional network strength and gene expression patterns ^12^. Distinct PLS-R models were separately developed for old and young groups. Each model utilized a predictor matrix (100 × 12,506) comprising AHBA's regional gene expression data, against a response vector of 100 elements representing the magnitude of SD-induced RNS alterations in the left cortical area, originating from one of the two groups. The PLS1 represented a linear composite of gene expression scores, weighted to reflect their collective covariance with alterations in RNS most closely. Spatial permutation test was used to validate the variance explained by PLS1. A bootstrapping technique was employed to calculate the Z-scores for each gene as their contributions to the PLS1 ^13^. The analytical code for this process was adapted from a publicly available resource on GitHub provided by Sarah Morgan ^12^, available at https://github.com/SarahMorgan/Morphometric_Similarity_SZ.

#### 3.3　|　Gene Category enrichment analysis

For statistically significant PLS models, the resulting ranked gene lists (Z-score > 3 or < -3, *p*_FDR_ < 0.05) ^12,14^ were tested for overrepresentation of gene ontology (GO) terms using the gene category enrichment analysis tool ^15^ (available at https://github.com/benfulcher/GeneCategoryEnrichmentAnalysis). Biological process categories are associated with specific subsets of genes as annotated in the Gene Ontology (geneontology.org). For each significant PLS model, the score for an enriched category was calculated as the mean gene Z-score in the bootstrapping procedure within the category. A null model was created by permuting the response variable 10,000 times, ensuring the maintenance of spatial autocorrelation using the spatial permutation test. Following this, PLS analysis was reapplied to the original gene expression matrix and the permuted response variables, allowing for the recalculation of null genes and enriching the null GO category biological process. The significance p-value (*p*_spin_) for each GO category was calculated based on its occurrence frequency relative to the permuting number. A significant level was set at *p*_spin_ < 0.05.


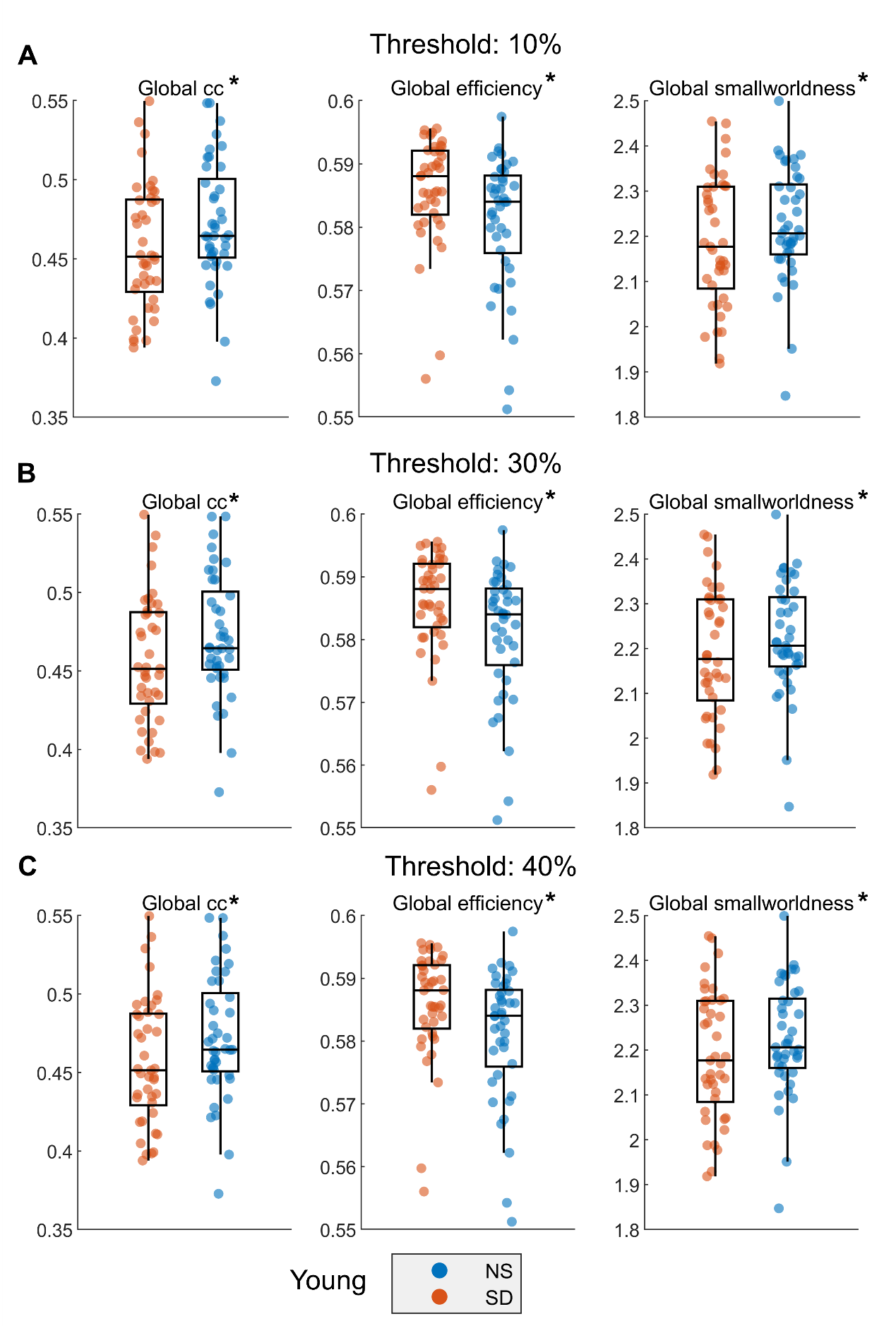


**Figure S1** Within-group differences with alternative thresholds (10%, 30%, 40%) of the global graph measures in the young group. *, pFDR < 0.05. Abbreviations: cc, clustering coefficient; NS, normal sleep; SD, sleep deprivation.

**Table S1. Demographic data**

| **Variables** | **Young (n = 47)** | **Old (n = 39)** |
| --- | --- | --- |
| **Demographics** |  |  |
| Sex, female (%) | 24 (51.1%) | 20 (51.3%) |
| Age (median, interquartile range) | 23.0 (21.5, 25.0) | 68.0 (67.0, 71.0) |
| Body Mass Index | 22.9 (3.1) | 24.7 (3.4) |
| **Hospital Anxiety and Depression Scale** |  |  |
| Depression | 1.1 (1.4) | 1.2 (1.0) |
| Anxiety | 2.8 (2.4) | 1.4 (1.4) |
| **Sleep** |  |  |
| Insomnia severity index | 10.6 (2.1) | 9.3 (1.7) |
| Karolinska Sleepiness Scale, full sleep | 5.9 (1.8) | 4.5 (1.8) |
| Karolinska Sleepiness Scale, sleep restriction | 7.7 (1.4) | 5.8 (1.7) |
| Total sleep time (min), full sleep | 429.1 (77.4) | 388.9 (68.3) |
| Total sleep time (min), sleep restriction | 185.3 (36.7) | 158.9 (31.9) |
| REM sleep (min), full sleep | 86.8 (29.9) | 74.9 (35.6) |
| REM sleep (min), sleep restriction | 28.2 (15.8) | 25.7 (18.7) |
| Slow wave sleep (min), full sleep | 98.0 (32.0) | 41.1 (33.1) |
| Slow wave sleep (min), sleep restriction | 70.5 (16.5) | 27.2 (24.4) |

*Note:* Continuous values are reported as means with standard deviations, unless otherwise indicated. Categorical data are reported with percentages. Sleep measures are reported in minutes.

**Table S2 Partial sleep deprivation effects on regional network strength across old and young group**

| **Group** | **Region** | **t-value** | ***p*** | ***p*_FDR_** |
| --- | --- | --- | --- | --- |
| **Young** | LH_Vis_5 | 3.494 | 0.001 | 0.043 |
|  | LH_Default_Par_4 | -4.092 | 0.000 | 0.022 |
|  | LH_Default_PFC_2 | -3.449 | 0.002 | 0.049 |
|  | LH_Default_PFC_4 | -3.519 | 0.001 | 0.043 |
|  | LH_Default_pCunPCC_2 | -3.536 | 0.001 | 0.043 |
| **Old** | LH_Vis_4 | 3.575 | 0.001 | 0.047 |
|  | LH_Default_pCunPCC_1 | -3.771 | 0.000 | 0.037 |
|  | RH_DorsAttn_Post_7 | -3.911 | 0.000 | 0.037 |

Note: The table provides information for statistically significant regions, including anatomical labels, paired t-test t-statistics, and p-values. All p-values are <0.05, FDR corrected. Each region name are derived from the Schaefer 200 functional parcellation ^11^, which can be found in the website: <https://github.com/PennLINC/AtlasPack/blob/main/Schaefer/atlas-Schaefer2018v0143_desc-200ParcelsAllNetworks_dseg.tsv>. Abbreviations: LH, Left hemisphere; R–Right. Cont=Control; DorsAttn=Dorsal Attention; SalVentAttn=Salience-Ventral Attention; SomMot=Somato-Motor; Vis=Visual.

**Table S3. Between-group differences in graph measures at baseline and after sleep deprivation**

| **Variables** | **Young (n = 41)** | **Old (n = 36)** | **t-value** | ***p*** | ***p*_FDR_** |
| --- | --- | --- | --- | --- | --- |
| **Baseline** | | | | | |
| Average RNS | 43.574 (0.100) | 43.571 (0.084) | -0.159 | 0.874 | 0.879 |
| Global CC | 0.472 (0.039) | 0.463 (0.038) | -0.992 | 0.324 | 0.648 |
| Global efficiency | 0.581 (0.010) | 0.583 (0.008) | 0.694 | 0.490 | 0.784 |
| Global smallworld | 2.223 (0.124) | 2.190 (0.131) | -1.151 | 0.253 | 0.648 |
| **After sleep deprivation** | | | | | |
| Average RNS | 43.579 (0.060) | 43.576 (0.079) | -0.152 | 0.879 | 0.879 |
| Global cc | 0.457 (0.040) | 0.468 (0.047) | 1.081 | 0.283 | 0.648 |
| Global efficiency | 0.586 (0.009) | 0.581 (0.011) | -2.056 | 0.0437 | 0.350 |
| Global smallworld | 2.189 (0.143) | 2.204 (0.170) | 0.417 | 0.768 | 0.879 |

*Note:* Continuous values are reported as means with standard deviations, unless otherwise indicated. RNS, regional network strength; CC, clustering coefficient; FDR, False Discovery Rate.

References:

1. Nilsonne, G. *et al.* Intrinsic brain connectivity after partial sleep deprivation in young and older adults: results from the Stockholm Sleepy Brain study. *Sci. Rep.* **7**, 9422 (2017).

2. Bastien, C. Validation of the Insomnia Severity Index as an outcome measure for insomnia research. *Sleep Med.* **2**, 297–307 (2001).

3. Lisspers, J., Nygren, A. & Söderman, E. Hospital Anxiety and Depression Scale (HAD): some psychometric data for a Swedish sample. *Acta Psychiatr. Scand.* **96**, 281–286 (1997).

4. Nordin, M., Åkerstedt, T. & Nordin, S. Psychometric evaluation and normative data for the Karolinska Sleep Questionnaire. *Sleep Biol. Rhythms* **11**, 216–226 (2013).

5. Åkerstedt, T. *et al.* Effects of late-night short-sleep on in-home polysomnography: relation to adult age and sex. *J. Sleep Res.* **27**, e12626 (2018).

6. Tamm, S. *et al.* A combined fMRI and EMG study of emotional contagion following partial sleep deprivation in young and older humans. *Sci. Rep.* **10**, 17944 (2020).

7. Anderer, P. *et al.* An E-health solution for automatic sleep classification according to Rechtschaffen and Kales: validation study of the Somnolyzer 24 x 7 utilizing the Siesta database. *Neuropsychobiology* **51**, 115–133 (2005).

8. Tamm, S. *et al.* The effect of sleep restriction on empathy for pain: An fMRI study in younger and older adults. *Sci. Rep.* **7**, 12236 (2017).

9. Hawrylycz, M. J. *et al.* An anatomically comprehensive atlas of the adult human brain transcriptome. *Nature* **489**, 391–399 (2012).

10. Markello, R. D. *et al.* Standardizing workflows in imaging transcriptomics with the abagen toolbox. *eLife* **10**, e72129 (2021).

11. Schaefer, A. *et al.* Local-Global Parcellation of the Human Cerebral Cortex from Intrinsic Functional Connectivity MRI. *Cerebral Cortex* **28**, 3095–3114 (2018).

12. Morgan, S. E. *et al.* Cortical patterning of abnormal morphometric similarity in psychosis is associated with brain expression of schizophrenia-related genes. *Proceedings of the National Academy of Sciences* **116**, 9604–9609 (2019).

13. Whitaker, K. J. *et al.* Adolescence is associated with genomically patterned consolidation of the hubs of the human brain connectome. *Proc. Natl. Acad. Sci. U.S.A.* **113**, 9105–9110 (2016).

14. Martins, D. *et al.* Transcriptional and cellular signatures of cortical morphometric remodelling in chronic pain. *Pain* **163**, e759–e773 (2022).

15. Fulcher, B. D., Arnatkeviciute, A. & Fornito, A. Overcoming false-positive gene-category enrichment in the analysis of spatially resolved transcriptomic brain atlas data. *Nat Commun* **12**, 2669 (2021).
